# Larval Physiological Responses to Temperature Across the European Distribution Range of a Global Invader at Home: The Shore Crab *Carcinus maenas*

**DOI:** 10.1002/ece3.71587

**Published:** 2025-06-13

**Authors:** Jan Phillipp Geißel, Noé Espinosa‐Novo, Luis Giménez, Nicole Aberle, Gro I. van der Meeren, Steffen Harzsch, Maarten Boersma, Gabriela Torres

**Affiliations:** ^1^ Alfred‐Wegener‐Institut, Helmholtz‐Zentrum für Polar‐ und Meeresforschung Biologische Anstalt Helgoland Helgoland Germany; ^2^ Department of Cytology and Evolutionary Biology, Zoological Institute and Museum University of Greifswald Greifswald Germany; ^3^ School of Ocean Sciences, College of Environmental Sciences and Engineering Bangor University Menai Bridge UK; ^4^ Institute of Marine Ecosystem and Fishery Science (IMF) University of Hamburg Hamburg Germany; ^5^ Department of Biology, Trondhjem Biological Station Norwegian University of Science and Technology (NTNU) Trondheim Norway; ^6^ Institute of Marine Research Austevoll Research Station Storebø Norway; ^7^ Alfred‐Wegener‐Institut, Helmholtz‐Zentrum für Polar‐ und Meeresforschung Wattenmeerstation Sylt Sylt Germany; ^8^ University of Bremen Bremen Germany

**Keywords:** intraspecific trait variation, larval performance, latitudinal variation, phenotypic physiological plasticity, thermal tolerance

## Abstract

In marine species with complex life cycles, thermal tolerance is usually narrower in larvae than in adults. Hence, range contraction and expansion, as a consequence of climate change, may be enhanced or hampered by among‐population variability in the thermal tolerance of larval stages. We quantified the performance (i.e., survival, development, and growth) of larvae of the shore crab *Carcinus maenas* at different temperatures (range 9°C to 27°C in steps of 3°C) in populations located towards the limits of the European distribution range (South: Vigo, Spain; North: Bergen and Trondheim, Norway). We hypothesised that, given the geographical distance, larvae from northern populations would show increased tolerance to low temperatures while those from southern populations would show increased tolerance to high temperatures. Such patterns would enhance poleward range expansion and counteract contraction as compared with a scenario where thermal tolerance does not change along the latitudinal gradient. Populations from southern Europe (Spain) showed slightly increased survival at higher temperatures compared to those further north and in invasive North American populations. However, there was little variation in larval tolerance between populations of Northern Spain and Norway: survival and growth rates were low at temperatures 9°C and 27°C. Larvae from the northernmost European populations (Norway) showed significantly shorter duration of development at low temperatures, which might have an adaptive value, contingent on the actual pattern of temperatures experienced during the larval phase. Further range expansions (or contractions) are likely to be driven solely by increasing temperatures unless populations located right at the range limit show increased tolerance to low (or high) temperatures.

## Graphical abstract

In larvae of the European shore crab *Carcinus maenas* , the temperature of highest survival decreased towards higher latitudes among populations distributed along the North European coast. *C. maenas* is native to Europe, but it is considered a global invader in coastal zones.

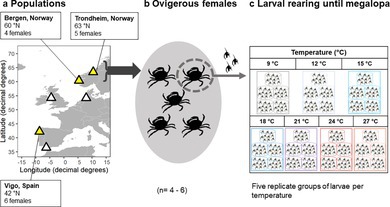

## 1 Introduction

Human‐induced climate change has caused severe impacts on marine ecosystems (Boersma et al. 2016; Burrows et al. 2011; Chan and Briski 2017; García Molinos et al. 2016; Gurevitch et al. 2011; Poloczanska et al. 2013). Temperature is one of the most important physical environmental factors driving growth rates of ectotherms, phenology, species distribution, population persistence, and community composition and diversity (Angilletta 2009; Genner et al. 2010). Particularly in the European seas, changes in species composition are expected as the water temperature has been rising two to three times faster than in the global average (Mackenzie and Schiedek 2007; Belkin 2009; Isaksen et al. 2022; de Amorim et al. 2023). In the southern parts of the European Atlantic coast, warming leads to community shifts termed “subtropicalization” (Montero‐Serra et al. 2015). Towards the equatorial side of the distribution range, species may retract poleward because the temperature is at or beyond the organisms' upper thermal limits or due to competition with warm‐adapted species arriving from lower latitudes (Souza et al. 2022; Sunday et al. 2012). Along the European North Atlantic coast, including the North Sea, range shifts related to ocean warming (Philippart et al. 2011) have been observed in, for example, plankton species (Beaugrand et al. 2009), demersal fish (Amelot et al. 2023), pelagic fish (Brander et al. 2003), and benthic rocky shore species (Mieszkowska et al. 2005). Towards the Arctic, the rise in sea surface temperature, the decline of sea ice, and the inflow of Atlantic water masses lead to the retraction of Arctic species northward and to deeper areas, and to the introduction of boreal Atlantic species (Jørgensen et al. 2022; Philippart et al. 2011); a process known as “Atlantification of the Arctic” (Kortsch et al. 2015; Wassmann 2011).

Intertidal ecosystems react in a particularly pronounced and rapid way in response to anthropogenic drivers (Helmuth, Broitman, et al. 2006; Helmuth, Mieszkowska, et al. 2006; Somero 2002). Many intertidal invertebrates develop through a complex life cycle, characterized by a pelagic larval phase (Levin and Bridges 1995; Pechenik 1999). Larvae are dispersive as they can drift in the water column for periods of variable duration. Depending on the temperature, most plankton‐feeding larvae require in the order of weeks to develop to a settling stage (Álvarez‐Noriega et al. 2020; McConaugha 1992; McEdward 1995; Shanks 2009). Given their dispersive nature, much of the natural process of changes in species distribution should occur through larval dispersal, driven by, for example, temperature and current patterns (Cowen et al. 2006; Cowen and Sponaugle 2009). In addition, planktonic larvae can be transported in great quantities in the ballast water of ships, hence contributing to the introduction and establishment in other habitats (Rilov and Crooks 2009; Verna et al. 2016; Ware et al. 2016; Williams et al. 1988) and to the connectivity among populations (Cowen and Sponaugle 2009). Given that the larval environmental tolerance range appears to be narrower than that of adults (Pandori and Sorte 2019), the range of larval thermal tolerance will set the limit to the capacity to endure increased temperature or establish new populations at the poleward distribution limits (Behrens Yamada et al. 2022; Carlton and Cohen 2003; Cohen et al. 1995; Cowen and Sponaugle 2009; Giménez et al. 2020).

*C. maenas* is native to the European Atlantic coast and is a well‐known global invasive species present on the shores of all continents except Antarctica (Leignel et al. 2014; Roman and Palumbi 2004; Young and Elliott 2020). It is an opportunistic omnivorous decapod crustacean and an ecosystem engineer (Klassen and Locke 2007). When introduced into new habitats, it may cause a restructuring of food webs (Cordone et al. 2023) by serving as a new food item for apex predators (Yorio et al. 2020) or by reducing the densities of many taxa as a predator (Grosholz and Ruiz 1995). *C. maenas* has a complex life cycle with four planktonic zoeal stages and a semi‐benthic megalopa stage at the end of the pelagic larval development (Williams 1967), followed by the benthic juvenile and adult crab stages. The pelagic larvae are the main dispersive stages, known to travel up to 200 km with regional tidal regimes, wind‐driven transport, and currents (Domingues et al. 2012). This dispersal distance depends on the duration of the pelagic phase, behavioral patterns, depth distribution, as well as local conditions like tidal currents or estuarine circulations (Corell et al. 2012; Moksnes et al. 2014; Shanks 2009). In North America, poleward range expansions of *C. maenas* are part of an ongoing biological invasion (Behrens Yamada et al. 2022; Cohen et al. 1995). In the European Arctic, potential expansions following warming and possibly local adaptations would make *C. maenas* a “neonative” species (Essl et al. 2019; Lohrer and Whitlatch 2002). One would also expect that increasing temperatures eventually result in a poleward shift of the equatorward range limit (García Molinos et al. 2016; Poloczanska et al. 2013, 2016).

Currently available information on the European shore crab, *C. maenas* , indicates that complete larval development is limited to temperatures > 9°C–12°C and is restricted to spring–summer in native and invaded environments (Dawirs 1985; deRivera et al. 2007). While larvae from North American populations do not appear to develop successfully to megalopa at temperatures above 22.5°C (deRivera et al. 2007), those from European populations complete the larval phase at temperatures as high as 24°C (Šargač et al. 2021, 2022; Spitzner et al. 2019). Hence, understanding changes in range distribution in coastal species will require quantification of larval thermal tolerance, especially towards their latitudinal distribution limits (both towards the poles and the equator). This need is highlighted by the fact that larval tolerance to environmental stressors appears to differ between core and edge populations (Geißel et al. 2024; Šargač et al. 2021). In *C. maenas*, a critical knowledge gap concerns the thermal range of survival and growth in larvae of northern European populations. This information is crucial to determine if low temperatures constrain successful larval development and future poleward range expansions. Most of the available information on the effect of temperature on survival and growth of European populations comes from local populations in the German Bight (Helgoland: 54°N Dawirs 1985, Spitzner et al. 2019), the Baltic Sea (Kerteminde: 55°N, Šargač et al. 2021) the Irish Sea, Isle of Man (54°N, Mohamedeen and Hartnoll 1989) and southern Spain (Cadíz: 36°N, Šargač et al. 2022) but *C. maenas* occurs from Mauritania (~20°N, Carlton and Cohen 2003 and references therein) to northern Norway (~70°N, GBIF 2023, collections from citizen scientists and communications with Norwegian scientists). Besides, the information available on thermal tolerance from larvae in the invaded range in North America comes from two populations: one located on the Atlantic and one on the Pacific coast (deRivera et al. 2007). Invasive populations of *C. maenas* in North America were only able to expand their range northwards after strong “El Niño” events on the West coast via larval transportation from the south (Behrens Yamada et al. 2022). On the East US coast, *C. maenas* poleward range expansions occurred due to invasion events with larvae originating from the northern European populations; the latter appear to be more tolerant to cold temperatures than those found previously on the East US coast (Roman and Darling 2007). Yet, information on the thermal tolerance over a wide latitudinal range is central to better assess the potential to cope with increased temperatures towards the equator and for poleward expansion, as well as invasion potential worldwide. A comparison of populations from Germany and one located near the southern European distribution limit (Cádiz: Šargač et al. 2022) has highlighted a diversity of larval responses to temperature, likely to contribute to the potential of invasion of coastal areas across the globe.

**FIGURE 1:**
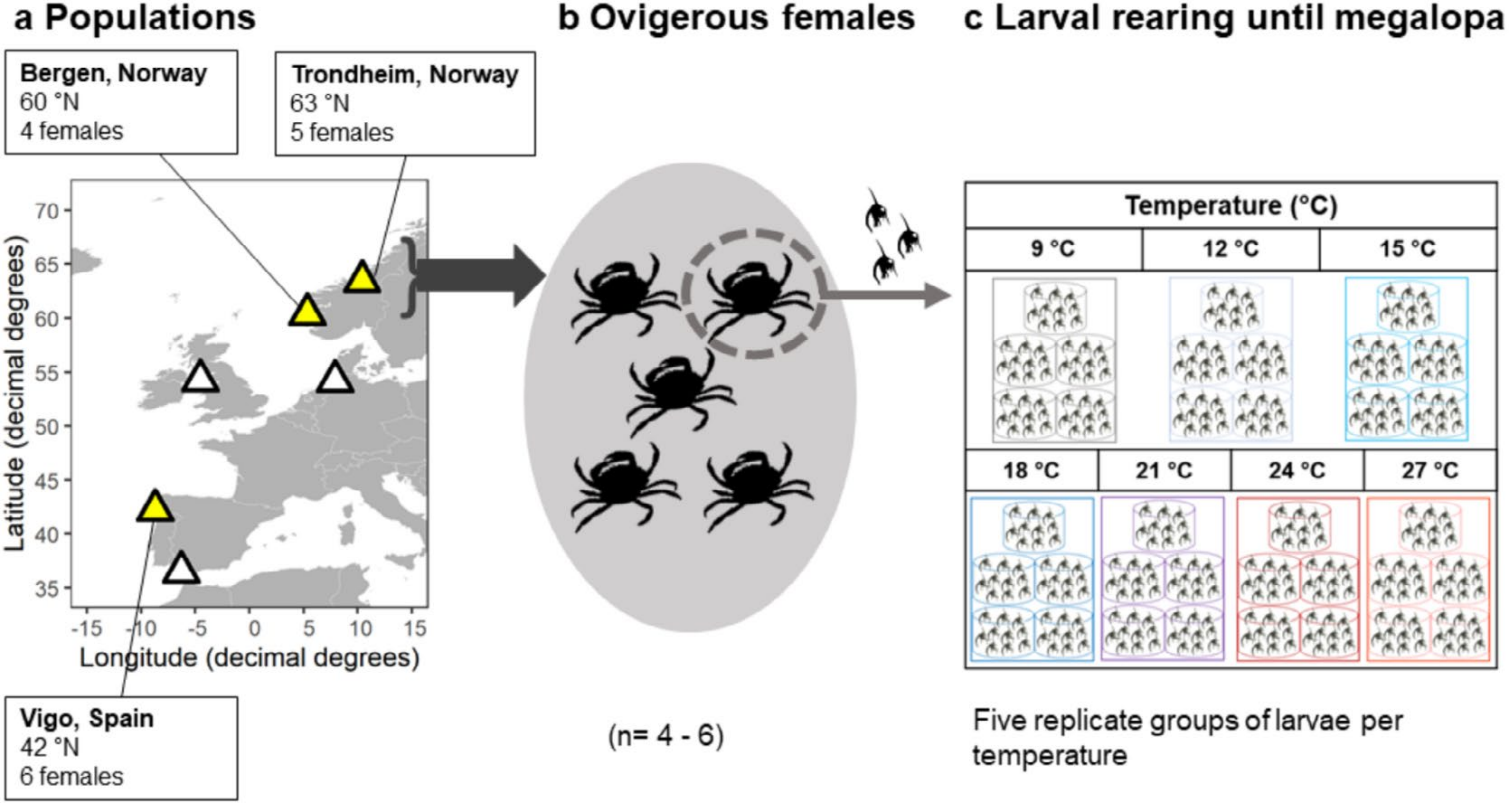
(a) Map illustrating the collection sites along the European Atlantic coast. Collection sites are shown by yellow triangles, white triangles show sites where literature data is available: German Bight, Helgoland; Irish Sea, Isle of Man; Atlantic coast in south Spain, Cádiz (Dawirs 1985; Mohamedeen and Hartnoll 1989; Šargač et al. 2022). (b) Experimental design to study the responses of larvae of *Carcinus maenas* from three different populations: Vigo (Spain), Bergen, and Trondheim (Norway), to different temperature conditions. Experiments were repeated using larvae originating from different females aiming at *n* ≥ 5 (Vigo: n = 6, Bergen: *n* = 4, and Trondheim: *n* = 5). (c) Larvae of *C. maenas* were reared from hatching to megalopa at seven different temperatures: 9°C, 12°C, 15°C, 18°C, 21°C, 24°C, and 27°C, represented in the picture from light grey (9°C) to red (27°C). Larvae were reared in 5 replicate rearing beakers of 10 individuals each.

The objective of this study was to quantify the thermal performance of *C. maenas* larvae (survival, development, and growth) over a wide latitudinal range (42°N to 63°N latitude) to complement already published data on populations in southern Spain and Germany (Šargač et al. 2022). We carried out a study of two populations from Norway (Bergen and Trondheim) in order to extend the information regarding tolerances towards the north of the native distribution. We also studied an additional population from northern Spain (Vigo) in order to have a better coverage of the responses towards the southern limit of the European distribution range. We compared our results with the available data from European waters in the German Bight (Helgoland), the Irish Sea (Isle of Man), and southern Spain (Cádiz) (Dawirs 1985; Mohamedeen and Hartnoll 1989; Šargač et al. 2022, and unpublished data), to obtain a more detailed description of early larval traits along a wide latitudinal gradient in the native environment (Figure 1). For comparisons, we also included data from two populations from North America (deRivera et al. 2007). Given the geographical distance between the southern and the northern populations, we hypothesize that the northernmost populations should show increased tolerance to low temperatures while the southern populations should show increased tolerance to high temperatures.

## 2 Materials and Methods

### 2.1 Collection and Maintenance of Females

Ovigerous females of *Carcinus maenas* (carapace length: 29.0–62.5 mm) were collected during their reproductive season, from three locations along their distribution range along the European coast (Figure 1a): Vigo (Spain, coordinates: 42°07′08.4″N, 8°49′19.0″W), Bergen (Austevoll, Norway, coordinates: 60°07′58″N, 5°05′35″E) and Trondheim (Norway, coordinates: 63°26′24″N, 10°24′0″E). The range of average monthly sea surface temperatures of the chosen sites are as follows: Vigo, 12°C–19°C; Bergen, 2°C–16°C; Trondheim, 3°C–15°C (averages calculated for the years 2012 to 2022 using the E.U. Copernicus Marine Service Information; https://doi.org/10.48670/moi‐00165). The temperatures measured at time of collection were: Vigo, 15°C; Bergen, 12°C; Trondheim, 12°C. Females from the Vigo population were hand‐collected during low tide by divers in the intertidal/subtidal zone of the mouth of the Miñor river; and transported to the Estación de Ciencias Mariñas de Toralla (ECIMAT). Females from Bergen were hand‐collected during low tide on beaches and rocky shores across the Austevoll archipelago and transported to the aquarium facilities of the Institute of Marine Research (IMR), Austevoll Research Station. In Trondheim, females were hand‐collected during low tide along the southern shoreline of Trondheimsfjord and at the entrance to the fjord close to Sletvik field station, Department of Biology, Norwegian University of Science and Technology (NTNU). These two locations were known, from preliminary observations, to harbour shore crabs in dense populations, which ensured the availability of ovigerous females during the reproductive season. Prior to the transport to the Biologische Anstalt Helgoland at Alfred‐Wegener‐Institute, Helgoland, Germany (Alfred‐Wegener‐Institut Helmholtz‐Zentrum für Polar‐und Meeresforschung 2023), ovigerous females were kept in flow‐through systems using local natural seawater (water temperature and salinity corresponding to their respective sampling site, range 32‰–35‰). For the transport to Germany, animals were individually placed in plastic containers (1 L), partially filled with water from their respective sampling site and a wet towel. The containers were then placed in a Coleman cooler box to ensure constant temperatures during transportation. Upon arrival in the laboratory on Helgoland, females from each population were placed in individual aerated aquaria with natural UV‐treated and filtered (mesh size: 2 μm) seawater from Helgoland (salinity 33‰ ± 1‰) at the temperatures recorded at the time of collection in each site (Vigo: 15°C, Bergen and Trondheim: 12°C). Animals were fed with shrimps ( *Crangon crangon* ) twice per week and water was changed daily to ensure high water quality at hatching.

We tested if larval performance (survival, duration of development, and body mass) was affected by the maintenance time of ovigerous females in the laboratory. Experiments were carried out with larvae that had hatched between 4 and 35 days upon collection. We did not use larvae hatching immediately after collection (i.e., arrival to Helgoland) in order to reduce the effects of the collection and transport‐induced stress. Moreover, we only used larvae that hatched within 35 days upon collection to reduce potential effects of laboratory rearing.

### 2.2 Experimental Set‐Up and Larval Rearing

Larval performance (i.e., survival, duration of development, and growth rates) was quantified in individuals reared from hatching to megalopa at seven different temperatures, from 9°C to 27°C at 3°C intervals (Figure 1b). For each population, we repeated each experiment with larvae from different females (number of females: Vigo = 6; Bergen = 4; Trondheim = 5). Freshly hatched larvae obtained from each separate female were distributed into five replicate rearing beakers per experimental temperature (10 larvae per rearing beaker, total = 35 beakers distributed in 7 temperatures, Table 1). This experimental design allowed for the evaluation of the effects of female, population of origin, and temperature during larval development. The experimental temperatures were chosen to match the span of conditions the larvae could experience in their respective natural environments, and also extreme temperatures, to obtain the full response pattern across a wide temperature range (Collins et al. 2022).

**TABLE 1:** Statements of replication.

Scale of inference	Scale at which the factor of interest is applied	Number of replicates at the appropriate scale
		Female of origin
Population	Replicate rearing beaker within female of origin	5 females Trondheim 4 females Bergen 6 females Vigo

Larval rearing was performed following standard rearing techniques (Torres et al. 2021). Experiments were conducted in 60 mL rearing beakers (10 larvae / 60 mL, density = 1 individual per 6 mL) placed in temperature‐controlled rooms (±1°C) with a 12:12 h light: dark cycle. UV‐treated and filtered (mesh size: 2 μm) natural seawater (salinity 33‰ ± 1‰) was used in the experiments. During the daily water change, larvae were checked for moults (i.e., exuviates) and dead organisms, which were recorded to assess survival and duration of development. Exuviates and dead organisms were discarded. Larvae were fed *ad libitum* with fresh *Artemia* sp. in a concentration of ~5 nauplii/ml (Great Salt Lake Artemia).

Body mass, elemental composition (C and N content), and growth rates of larvae were quantified from samples taken at the start and at the end of each experiment. In crustaceans, larval carbon content is considered a proxy for lipid reserves, which is more sensitive to environmental variation than nitrogen content, a proxy for proteins (Anger and Harms 1990; Dawirs 1986; Dawirs et al. 1986). At the start of the experiment, freshly hatched zoeae I (5 replicates of 50 larvae each) were sampled within 24 h after hatching. At the end of the experiment, freshly metamorphosed megalopae were sampled within 24 h after metamorphosis. Larvae were gently pipetted onto a filter and rinsed with distilled water, carefully blotted dry with paper, and stored for analysis in pre‐weighted tin cups at −20°C. Prior to the elemental composition analysis and in order to determine dry mass, samples were freeze‐dried (for 48 h, Christ Alpha 1–4 freeze drier) and weighed (microbalance, Sartorius MCA2.7S‐2S00‐M, precision 1 μg). Each sample consisted of pooled larvae (i.e., 50 zoea I or 1–3 megalopa, total number of 1020 megalopae) and was weighted to reach the necessary minimum weight to run the C and N content measurements. Elemental composition (carbon and nitrogen content) was determined using an elemental analyser (vario MICRO cube CHNS analyser, Elementar Analysensysteme).

### 2.3 Data Analysis

We calculated survival to each zoeal stage as the percentage of survivors relative to the number of organisms at the beginning of each experiment (i.e., 10 larvae per rearing beaker). Duration of development to each larval stage was calculated as the time required to reach the next developmental stage, considering the duration of development of the previous stages. Instantaneous growth rates over the whole larval period were calculated as G = log (W_f_ / W_0_)/t, where W_0_ is the average mass (dry mass, carbon, or nitrogen) at hatching, W_f_ is the corresponding mass of each megalopa collected in each rearing replicate, and t is the time it took each corresponding larva to reach the megalopa stage.

The combined effects of temperature and population of origin were quantified using mixed modeling (Zuur et al. 2009). The response variables were survival, duration of development, elemental composition, and growth rates. The models consisted of temperature and population of origin as fixed factors, and female of origin as a random factor. We also explored correlations between female body size and larval performance (survival, duration of development, and growth rates), but did not find any evidence of female size explaining performance (Table S1); these results are in line with a previous study on *C. maenas* (Torres et al. 2020), where larval survival did not correlate with female size. We, therefore, did not consider female size as a predictor in any subsequent test (Table S1). Due to the orthogonal design of the experiment, we did not expect incubation times to affect the results regarding the effect of temperature on descriptors of larval performance. Yet, variations in incubation times could drive population effects. Hence, we used Pearson correlation to test if incubation times explained variation in larval performance for each combination of temperature and population. We did not find any evidence of such an effect (except for a few cases, correlations were not significant: Table S2); therefore, we did not consider incubation times as a response variable in the data analysis.

Model analysis was performed applying a backward model selection (Zuur et al. 2009) based on the second‐order Akaike information criterion (AICc). The package “nmle” (function lme and gls, Pinheiro et al. 2019; R Core Team 2022) was used for model fitting with generalized least squares. Selection of models was performed following two steps; in the first step, the random terms were compared through Restricted Maximum Likelihood (REML) and in the second step the fixed terms were compared through Maximum Likelihood, after refitting the model with the best random structure. When ΔAICc was ≥ 3, the model with the lowest AICc was always selected irrespective of complexity. When ΔAICc was < 3, the less complex model was selected if such a model had the lower AICc. However, if ΔAICc was < 3 and the most complex model had the lowest AICc, hypothesis testing (likelihood‐ratio tests, LRT) was used to test for model selection. In such a case: (i) when the models differed significantly (*p* < 0.05) we chose the model with the lowest AICc, and (ii) when the difference was not significant, we chose the less complex model. For survival analysis, we used the whole temperature range (from 9°C to 27°C), but for the rest of the statistical analysis we only used the results from 12°C to 24°C, as at 9°C and 27°C only a few larvae reached the megalopa stage. For dry mass, carbon and nitrogen content, the model did not retain latitude (ΔAICc < 3, Table S5). However, for duration of development, the model containing latitude was retained and thus we presented this trend in the results (ΔAICc > 3, Table S4).

Survival proportions were transformed to logistic (Warton and Hui 2011) and logarithmic scales, and data were analysed in both scales. We used the logarithmic transformation to test the multiplicative effect as a null model and the multiplicative model to test if temperature and population of origin have independent effects on the rates of survival (Piggott et al. 2015; Torres and Giménez 2020). Prior to the transformations, survival proportions were rescaled to avoid log(0) values; rescaled proportions were computed as *p*′ = [*p* (*n* − 1) + 0.5]/*n*, where n is the number of larvae at the beginning of the experiment (*n* = 10 individuals). We analysed the duration of development following two approaches: in the first one, we treated temperature as a factor; in the second approach, temperature was analysed as a continuous variable in order to fit functional relationships between temperature and duration of development.

We combined information available from the literature with the experimental data obtained in this study to quantify the changes in thermal tolerance along the native distribution range of *C. maenas* (Dawirs 1985; Mohamedeen and Hartnoll 1989; Šargač et al. 2022). In addition, we used available information from the North American coast (deRivera et al. 2007) in order to get a better idea of the range of thermal tolerance in invaded and native habitats. We gathered data on the temperature of optimal survival, lowest and highest temperatures that yield survival rates > 5%, and the duration of development (in 12°C and 15°C). We excluded the data point at 12.5°C for Helgoland in the data analysis of the temperature of optimal survival since it was based on experiments without replication at the level of “female of origin”, which meant no repetition of the experimental procedure using many females (Dawirs 1985). In order to quantify the effect of latitude, average sea surface temperature (SST, see Figure S1) of the reproductive period (days of SSTs between 9°C and 27°C) and average season length of the reproductive period (number of consecutive days of SSTs between 9°C and 27°C, see Figure S2) on the predictors for larval performance, we used mixed modelling (Zuur et al. 2009). We performed a backward model selection approach fixing a linear model. SSTs were derived from E.U. Copernicus Marine Service Information (https://doi.org/10.48670/moi‐00165) and averages per site were calculated from yearly averages based on daily SSTs for the years 2012 to 2022 after trimming the data to the relevant seasons within the temperature range (temperature between 9°C and 27°C).

## 3 Results

### 3.1 Survival, Duration of Development, and Growth Rates to Megalopa

**FIGURE 2:**
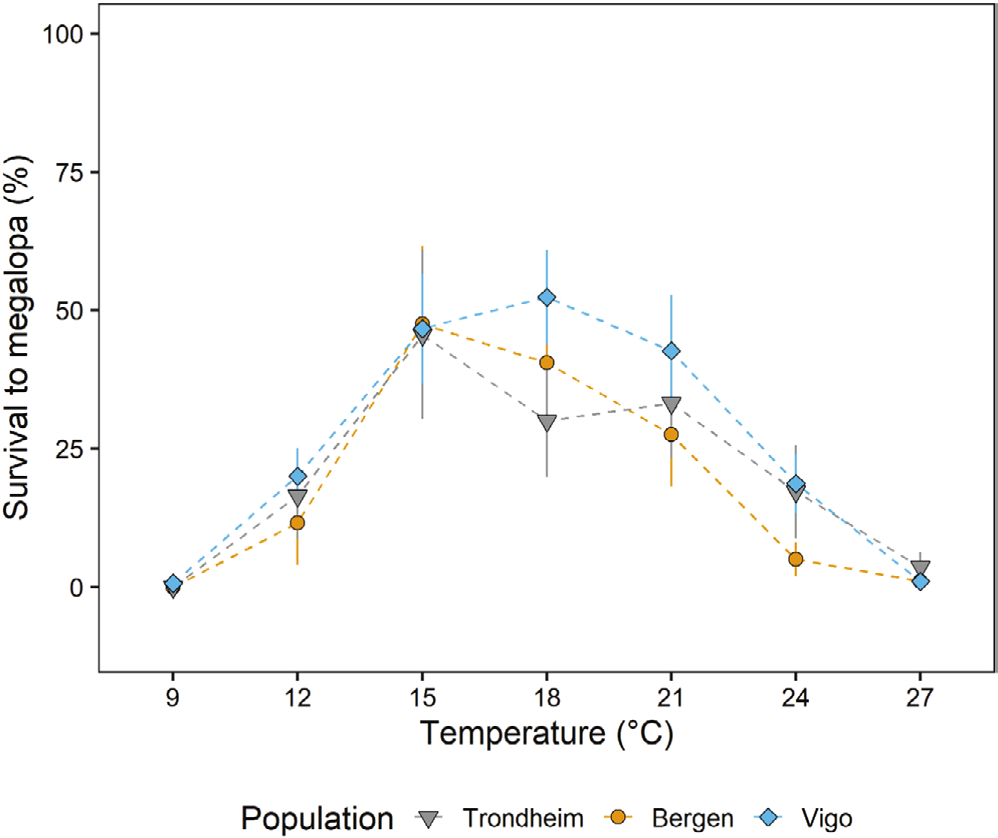
Average survival from hatching to megalopa of *Carcinus maenas* larvae reared under different temperatures, originating from females of three populations: Vigo, Bergen, and Trondheim. Data presented as mean values ± SE among larvae from different females within each population (*n* = 6 for Vigo; *n* = 4 for Bergen, and *n* = 5 for Trondheim). At 9°C and 27°C, statistical tests were not performed due to the low number of data points.

We did not find evidence of variations in larval survival to megalopa among populations (Figure 2 and Table S3); survival depended on temperature and varied among females (best model retained temperature in the fixed structure). For all three populations, only one larva metamorphosed to megalopa at 9°C (from Vigo). In all populations, survival was consistently low at 27°C. In the range of 12°C–24°C, there was some variation among populations, but it was overridden by the variability in survival among larvae from different females (Figure 2: note size of error bars). From 12°C, survival increased with temperature and reached a maximum in the range 15°C–21°C. Survival then decreased at higher temperatures, and low survival rates were recorded at temperatures ≥ 24°C for all three populations (Figure 2).

**FIGURE 3:**
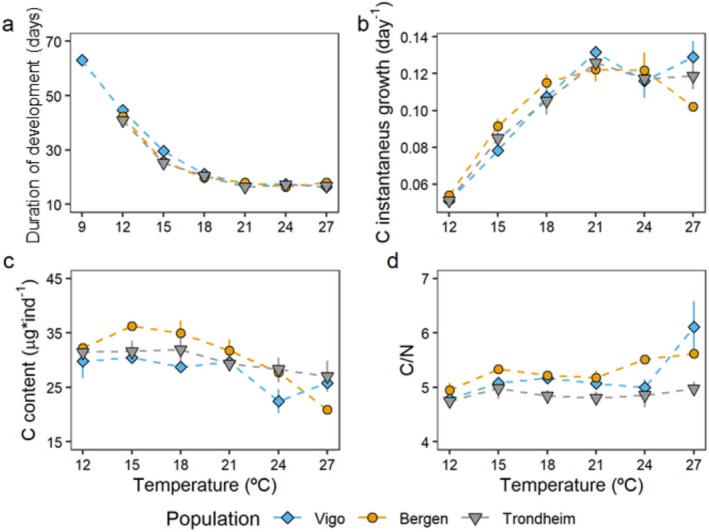
Effect of temperature on duration of development, growth, and elemental composition of larvae of *Carcinus maenas* from three different populations. Average duration of development (a), carbon growth rates (b) from hatching to megalopa. Average carbon content (c) and C/N (d) ratio of megalopa. Data presented as mean values ± SE among larvae from different females within each population (*n* = 6 for Vigo; *n* = 4 for Bergen and *n* = 5 for Trondheim). Symbols as in Figure 2. At 27°C, tests were not performed for this temperature due to a low number of samples.

Duration of development to megalopa decreased with increasing temperatures in a non‐linear pattern (Figure 3a); best models retained the interaction of temperature and population, when temperature was treated as a factor (Table S4a) and as a continuous variable (Table S4b). The two Norwegian populations showed a shorter duration of development compared to Vigo at the colder temperatures (Figure S3). Duration of development reached a plateau in the range 21°C–27°C of 16–18 days for all three populations (Figure 3a).

**FIGURE 4:**
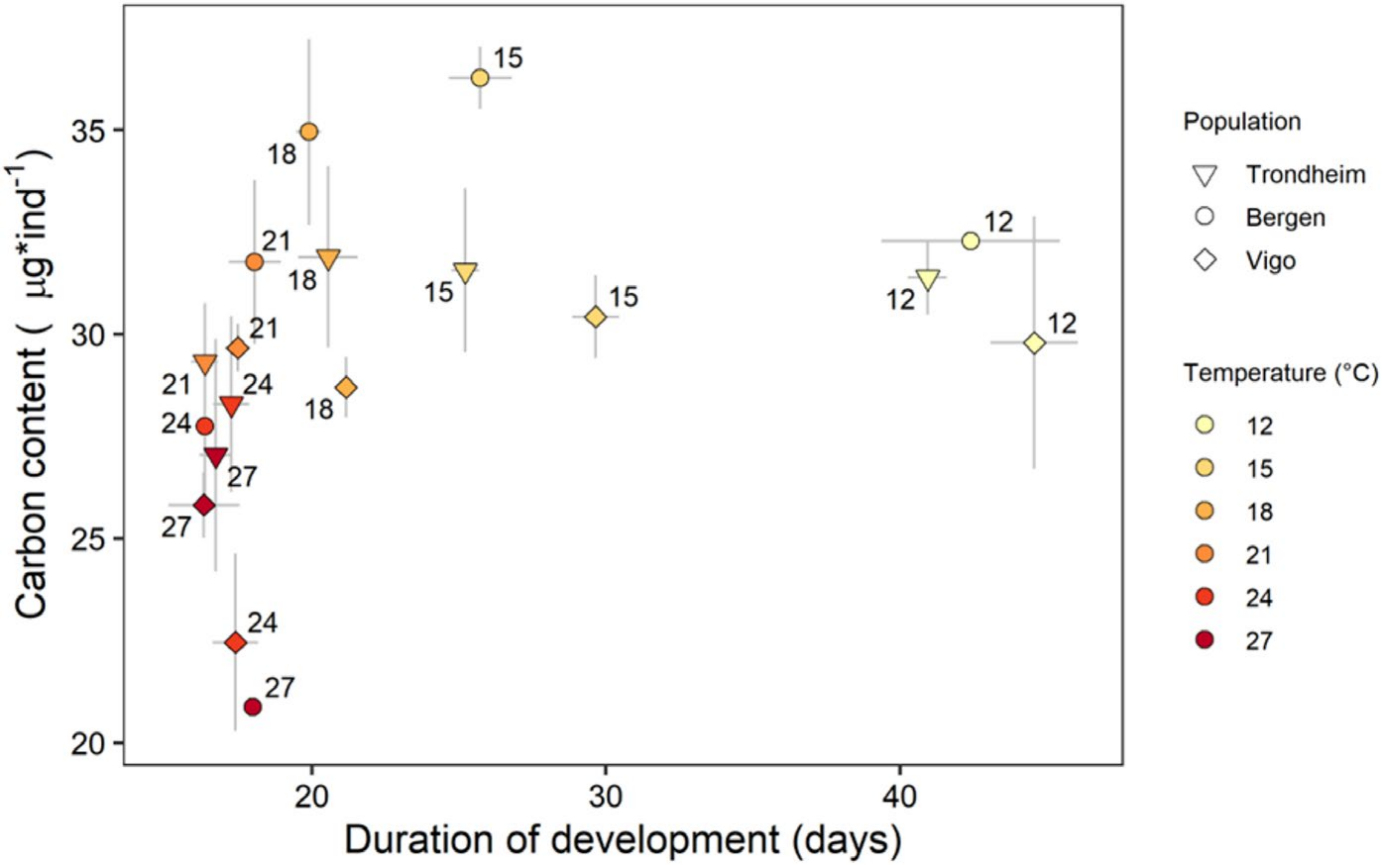
Integrated responses of carbon content and duration of development of larvae of *Carcinus maenas* reared under different temperatures, from hatching to megalopa, from larvae originating from females from three populations: Vigo, Bergen, and Trondheim. Data presented as mean values ± SE among larvae from different females within each population (*n* = 6 for Vigo; *n* = 4 for Bergen and *n* = 5 for Trondheim). Symbols: Vigo is represented by diamonds, Bergen with circles, and Trondheim with triangles. Colors and labels indicate temperature (°C).

Growth rates increased with temperature for the three populations up to 21°C when they reached a plateau (e.g., carbon content: Figure 3b and Figure S4b,d); the best model retained temperature, not population of origin, or any interactions (Table S5, 27°C not included in the test due to very low number of larvae). Growth rates in terms of dry mass and nitrogen content showed similar patterns to carbon content growth rates, that is, an increase up to 21°C (Table S5 and Figure S4b,d). For carbon content, the best model retained temperature and population operating in an additive way: individuals from Bergen contained more carbon than those from the two other locations (range 15°C–21°C). Carbon content showed some variations among temperatures; it was generally constant in the range 12°C–21°C, but it decreased at the highest temperatures (Figure 3c). Dry mass and nitrogen content showed similar patterns to the one described above, tending to decrease at higher temperatures (> 24°C, Figure S4a,c). Best models retained temperature and population operating in an additive way (Table S5). C/N ratios were generally constant in the range 12°C–24°C for all three populations (Figure 4d; best model retained population, Table S5).

Integrated growth responses to megalopa were characterised by a matching of duration of development and growth rates in the range of 12°C–21°C. In this range, the reductions in duration of development observed at higher temperatures (e.g., > 40 days at 12°C vs. 18 days at 21°C) coincided with increases in growth rates, leading to similar carbon content (30–36 μg*ind^−1^) and body mass at metamorphosis. By contrast, at 24°C and 27°C, reductions in duration of development were not matched by increases in growth rates (Figure 4) and larvae metamorphosed with reduced body mass. Integrated growth responses for dry mass and nitrogen showed similar patterns to those shown for carbon (Figure S5).

**FIGURE 5:**
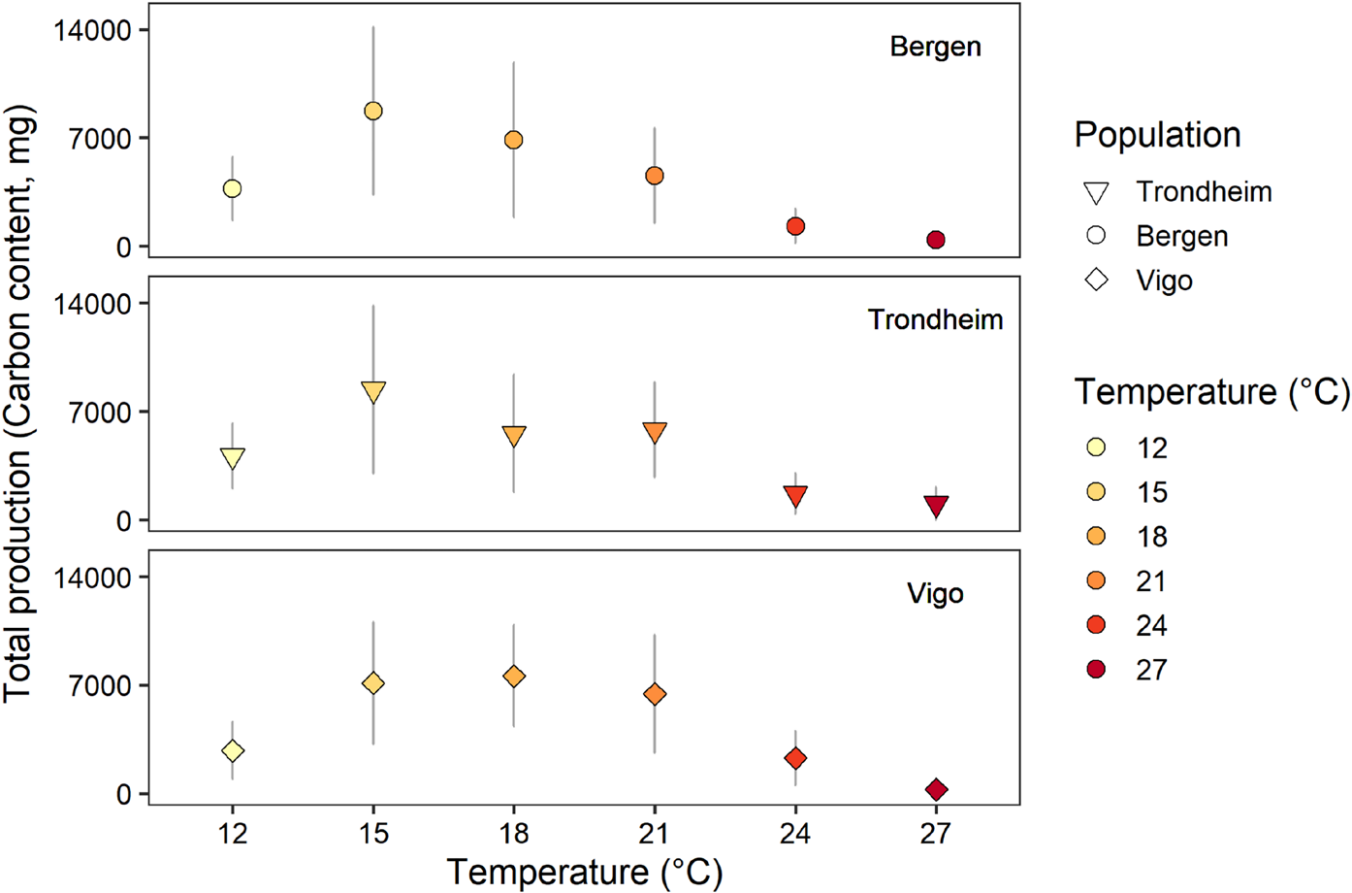
Effects of temperature in total production, measured as the number of survivors *carbon content of larvae of *Carcinus maenas* reared under different temperatures, from hatching to megalopa, from larvae originating from females from three populations: Vigo, Bergen, and Trondheim. Data presented as mean values ± SE among larvae from different females within each population (*n* = 6 for Vigo, *n* = 4 for Bergen, and *n* = 5 for Trondheim). Symbols as in Figure 4.

Total production in terms of the total carbon content of all surviving megalopae followed the same pattern as survival, with maximum values in the range 15°C–21°C for all three populations (Figure 5). Total production in terms of dry mass and nitrogen content followed the trend reported for survival as well, with maximum values in the range 15°C–21°C (Figure S6a,b).

### 3.2 Comparison of Results With Published Data

**FIGURE 6:**
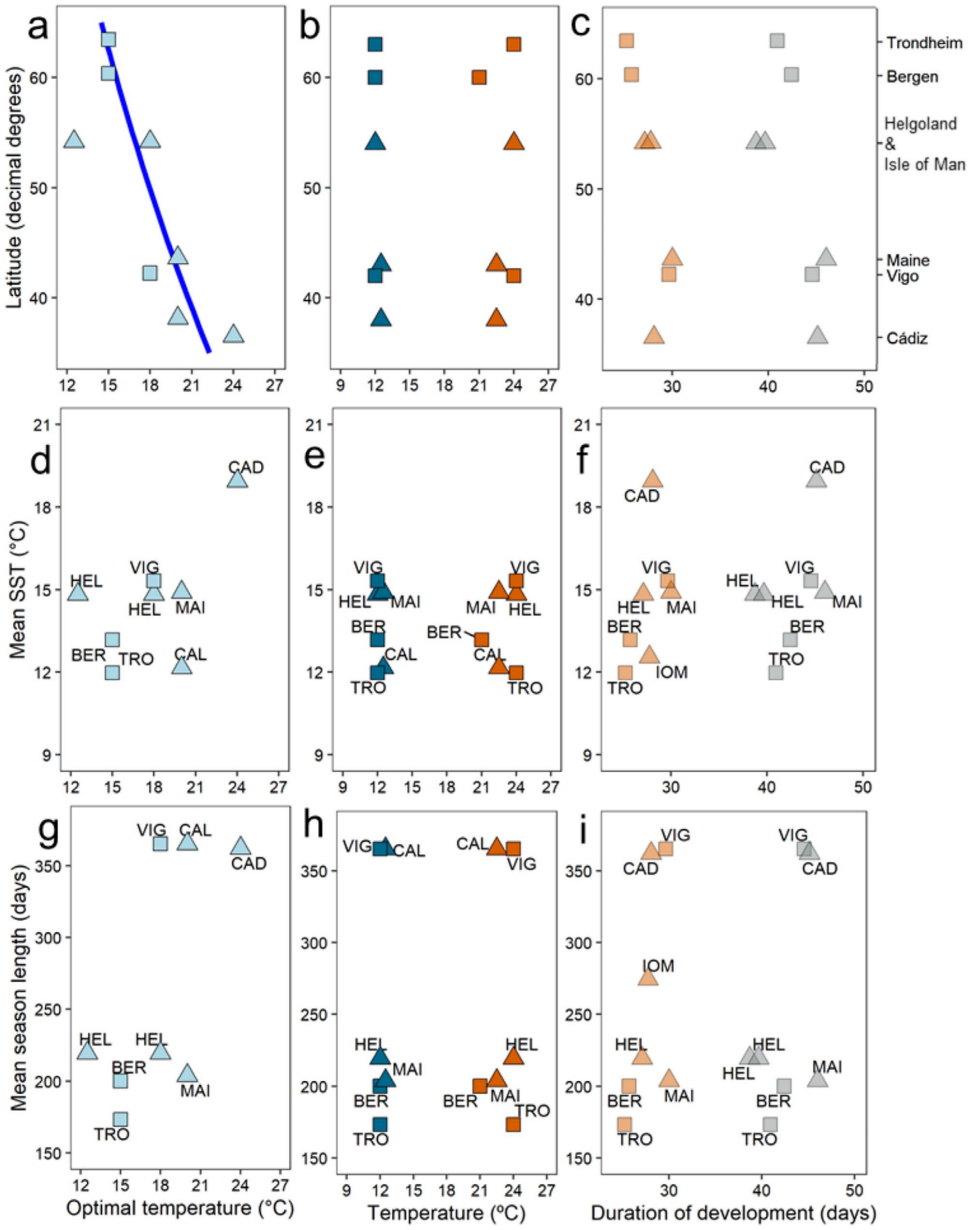
Temperature of highest survival rate (light blue symbols) by latitude (a), mean length of the season suitable for larval development, that is, days with SST between 9°C and 27°C (d), mean SST of the suitable season at the location of the population (g). Lowest (dark blue symbols) and highest (red symbols) tested temperatures at which *Carcinus maenas* larvae showed survival rates from hatching to megalopa > 5% by latitude (b), mean SST (e) and mean season length (h). Duration of development to megalopa (days) at 12°C (grey symbols) and 15°C (orange symbols) plotted by latitude (left y‐axis) and population (right y‐axis) (c), mean SST (f) and mean season length (i). In (a), the dark blue line indicates a general linear model; the best model retained latitude and was fitted using general linear model (ΔAICc = 4 vs. null model without latitude as predictor). In (a), the data point at 12.5°C (Helgoland) was not used in the data analysis, and there was no data available for Isle of Man. In (b), only temperatures are displayed for populations that have been tested in another more extreme temperature that did not yield survival. No data is available for the Isle of Man. Data points obtained in this study are shown as squares, and data from the literature are shown as upward triangles (Dawirs 1985; Mohamedeen and Hartnoll 1989; deRivera et al. 2007; Šargač et al. 2022, pers.com. Deschamps 2024). BER, Bergen; CAD, Cádiz; CAL, California; HEL, Helgoland; IOM, Isle of Man; MAI, Maine; TRO, Trondheim; VIG, Vigo.

We compared results of maximum survival during larval development and tolerance range obtained from this study with data already published for European and North American populations by latitude. We also tested if average SST and season length (number of consecutive days with temperature between 9°C and 27°C) would predict larval survival and tolerance. Not all data sets cover the thermal range (9°C–27°C) used in our experiments. For Cádiz, the range was 12°C–24°C; and we included the maximum survival recorded, which occurred at 24°C (Šargač et al. 2022). For Helgoland, Dawirs (1985) recorded no survival at 6 and very low survival at 9°C, and recent experiments showed that survival at 27°C was very low (Deschamps pers.comm.). For the populations of North America (range 10°C–22.5°C), the upper and lower thermal tolerances were reported (deRivera et al. 2007). Given the available data, we found that populations located towards higher latitudes showed higher survival at lower temperatures compared to those located towards lower latitudes (Figure 6a). The best model retained latitude as a predictor (ΔAICc = 4 vs. null model without latitude as predictor). The lowest and highest tested temperatures that allowed larval survival until metamorphosis > 5% showed no latitudinal trend for the data available (Figure 6b) and neither with average SST nor season length (Figure 6e,h). Overall, duration of development to megalopa for the populations located at higher latitudes was slightly shorter at 12°C and 15°C than for populations located at lower latitudes, but there was only limited support by statistics (Figure 6c, for 15°C: ΔAICc = 1 versus null model without latitude as predictor, for 12°C ΔAICc of null model without latitude as predictor 2 points lower). We also tested the temperature of highest survival, lowest and highest tested temperatures that allowed survival > 5%, and duration of development with average seasonal temperature and average season length, but neither were retained in the best model.

## 4 Discussion

We found only slight differences in the performance of *C. maenas* larvae in response to temperature among populations located towards the southern (Spain) and northern (Norway) European distribution limits. Larvae from females collected in Norway developed faster at lower temperatures (12°C–18°C) than those from a population from Vigo (Northern Spain). Only a single larva survived to megalopa at 9°C (belonging to the Vigo population), and very few larvae reached megalopa at 27°C (Vigo 1%, Bergen 1% and Trondheim 3.6%). On average, for all three populations, the highest survival was found in the range 15°C–21°C. Contrary to what we hypothesized, larvae from Norway did not exhibit higher survival at low temperatures than those from Spain. If there was such a pattern, it was overridden by variation in survival among larvae from different females, as shown by wide error bars (Figure 2) and highlighted by the fact that the best models retained female in the random structure (Table S3). In what follows, we briefly discuss within‐population variation in larval performance and then focus on variations among populations.

### 4.1 Larval Performance: Variation Within Populations

Within‐population variation in survival among larvae from different females has been found in previous studies on invertebrate larvae (Applebaum et al. 2014; Carter et al. 2013; Spitzner et al. 2019). However, the implications of such variation have rarely been considered in light of global warming so far (Bolnick et al. 2011; Violle et al. 2012). The question arises as to whether such variation in survival is driven by genetic‐heritable variability or parental effects, that is, effects of parental environment and/or phenotype on the phenotype and resulting performance of the offspring (Marshall et al. 2008; Uller et al. 2013). In *C. maenas*, for example, effects of maternal environments like temperature, salinity, and seasonality have been shown to drive offspring traits like fatty acid composition, embryo volume, and survival rates (Fernández 2025; Rey et al. 2017; Torres et al. 2020). Under a global warming scenario, genetic‐heritable variation within the population should result in portfolio effects (Schindler et al. 2015). Portfolio effects describe aggregate systems that are often less volatile than their components because of statistical averaging between the respective components. In our case, populations would be buffered from the effects of temperature increase by a diversity of individual responses through selection of warm‐tolerant genotypes. The implications of such variation of environmental origin are difficult to predict as they strongly depend on the combination of two factors: (1) how parental conditions (e.g., temperature or another stressor) drive survival when larvae experience increased temperature, and (2) how parental and larval conditions covary in the field. For example, if increased parental temperature drives decreased larval performance (at high temperatures), and long warming periods affect both the parental and larval habitat, then strong reductions in larval survival are expected. Parental effects are important (Parker et al. 2017; Torres et al. 2020), but their actions depend on the covariation between the environmental conditions of the parental and larval habitats. In this sense, we need further research, including experiments based on a larger pool of females and quantifying the role of parental effects and the covariation among environmental stressors in both the benthic and pelagic habitats.

### 4.2 Larval Thermal Tolerance: Variation Among Populations

Our results do not give evidence of a strong change in the pattern of tolerance to low temperatures in the populations of *C. maenas* occurring at the northern limit of the distribution range. According to findings from population genetics (Roman and Palumbi 2004), the two Norwegian populations of *C. maenas* are genetically similar but differ from the Spanish population. In that study, the western Norwegian population showed roughly 1/3 of haplotypes from a boreal clade and 2/3 from two clades that are not attributed and partially shared with the southern European populations. In addition, populations from Northern Spain had mostly haplotypes from those two shared clades and a small fraction of haplotypes from a southern clade. However, to the best of our knowledge, there is no available information on the genetic composition of the northernmost Norwegian populations. Perhaps, larval tolerance to low temperature is higher in Arctic populations from the northern range edge between Tromsø and the North Cape (GBIF 2023, collections by citizen scientists and communications with Norwegian scientists) when compared to sub‐arctic and temperate populations of *C. maenas* . Further studies are needed to explore potential adaptations of the northernmost populations, including population genetic analyses.

Our findings of 10%–20% shortened duration of development to metamorphosis to megalopa at low temperatures (12°C–15°C) in larvae from Norway as compared to Vigo represent the only evidence for some level of an adaptive response in our study. This trend is consistent with previous studies on other European and North American populations (Figure 6c). A similar pattern was found in interspecific comparisons among closely related fish species (Sanford et al. 2006), sea stars (Hoegh‐Guldberg and Pearse 1995), and deep‐water lithodid crabs (Brown et al. 2018). We categorize this response as “adaptive” because such a reduction in the duration of development can be beneficial for populations (Gotthard and Nylin 1995), without implying that it is local adaptation (the demonstration of which requires multigenerational experiments in common garden settings and/or reciprocal transplantation (Dunphy et al. 2012; Jung et al. 2013)). Shorter pelagic larval duration can be advantageous (Pechenik 1999), especially in cold temperatures, because in higher latitudes, the window for optimal larval development is narrower than in lower latitudes (Levin and Bridges 1995). This is particularly important for plankton‐feeding crustacean larvae, which are characterized by a limited capacity to tolerate starvation periods, as observed in *C. maenas* (maximum of a few days and less than the duration of any single zoeal stage, Dawirs 1984). The ecological consequences of such a reduction of the temporal window of larval development can be crucial since this can translate into differences in larval survival. For example, assuming per capita larval mortality rates estimated for marine invertebrate larvae in the field (=0.14 *d* ^−1^; White et al. 2014) and an exponential reduction in the number of survivors as a function of time, a reduction of 10% in duration of development (e.g., from 44 to 40 days at 12°C) would result in an increase of 75% in survival to the settling stage (from 211 to 370 survivors out of 10^5^ larvae released). The shorter duration of development in larvae from the Norwegian populations did not result in a reduced dry mass or carbon content of the megalopa (Figure 3c and Figure S2a,c). In addition, faster‐developing larvae would settle earlier in the season, hence the resulting juveniles should experience a longer period of summer temperatures enhancing post‐metamorphic growth. Post‐metamorphic body mass and size serve as a predictor of post‐metamorphic performance with a positive correlation between body mass and performance (Pechenik 2006). However, at this stage, it is difficult to quantify how much advantage is given by the observed reduction in duration of development in the Norwegian populations. This evaluation requires further field studies monitoring larval hatching, development, and settlement in combination with modeling of larval phenology (see e.g., deRivera et al. 2007; Giménez et al. 2020).

As compared to larvae, adults can tolerate a wider range of temperatures (2°C to 37°C, Tepolt and Somero 2014; Rivers 2024) while larval development to megalopa only occurred from 9°C to 27°C in this study and from 10°C to 22.5°C for North American populations (deRivera et al. 2007). Since larval tolerance is more limited, the larval season must be restricted, for example, to spring/summer in the North and Norwegian Seas. By contrast, adults occur all year round, including in winter when temperatures go well below the lower tolerance limit observed in larvae. But adults appear to show stronger differences among populations in response to temperature than larvae. Tepolt and Somero (2014) found that adult crabs from Norway (60.6°N) experienced heart collapse at lower temperatures (lower CT_max_) and had lower heartbeat frequencies at low temperatures than those from the Iberian Peninsula (38.6°N). Tepolt and Palumbi (2020) studied frequencies of single nucleotide polymorphisms (SNPs) in six populations from North America and Europe. They found a cluster of SNPs that appeared to be transmitted as a unit and correlated positively with cold tolerance. In addition, Stein et al. (2023) found that adult crabs of *C. maenas* from Vigo (42.2°N) showed higher robustness in the neuronal activity driving the stomatogastric system towards a high‐temperature stimulus than those from Helgoland (51.1°N). However, acclimation can blur the differences among populations: for example, Tepolt and Somero (2014) found that 25°C short‐term acclimation significantly increased CT_max_ relative to 5°C acclimation in most tested populations. Likewise, Stein et al. (2023) found that a similar shift in the critical temperature at which neuronal circuit activity breaks down was achieved by long‐term acclimation. In larvae, no sign of population‐specific cold or heat tolerance that strong has been found in this study. An absence of local adaptation to temperature across a large latitudinal gradient in a seasonally occurring planktonic life stage has been attributed to restricted seasonal occurrence and therefore restricted temperature ranges experienced by larvae (Mitchell and Lampert 2000). In any case, from the larval perspective, poleward expansion does not appear to be enhanced in Norwegian populations showing increased larval tolerance to low temperatures unless this is restricted to populations located in the Arctic not studied here. In a recent study, Norwegian populations exhibited higher survival to the second zoea at 9°C than the Vigo population, and while survival at 6°C was limited for all populations, LT_50_ in 6°C increased from south to north (Geißel et al. 2025). But it remains to be studied whether similar traits could be found in an Arctic population until metamorphosis. However, poleward range expansion is likely to occur because of the increasing temperatures in light of global warming, which will enable the establishment of populations through larval dispersal and post‐metamorphic survival through the increased tolerance to temperature exhibited by the benthic stages (Tepolt and Somero 2014). Intensifying marine heatwaves in the Arctic (Gou et al. 2025) might facilitate the poleward movement of the range edge even more (Deschamps 2024). At the southern range edge, however, increases in mean temperatures and marine heatwaves would lead to the decline in *C. maenas* populations (Monteiro et al. 2023; Souza et al. 2022).

For the Vigo population, we did not find evidence of increased larval tolerance to high temperatures compared to those of Norway. Survival dropped at 27°C, while the growth rate dropped at temperatures > 21°C, leading to smaller body mass at metamorphosis. In populations located in the southern Iberian Peninsula, for example, Cádiz, tolerance to temperatures above 21°C might increase the opportunities for successful larval development during summer, because surface water temperatures can reach above 25°C (Pérez‐Miguel 2019) and high summer temperatures (≥ 23°C) appear to limit larval presence in the water column (Sprung 2001). This scenario might be less applicable to Vigo with lower mean SSTs, and comparably low annual SST fluctuations, but similar annual length of season suitable for larval development to Cádiz (Figure 6; Figures S5 and S6). Our results suggest similar capacities in larvae of the three studied populations of *C. maenas* to survive, develop, and grow under increased temperatures. Their temperatures of maximum survival were lower than the temperature of highest larval survival in the southernmost studied population (Cádiz, 24°C: Figure 6a,b). However, larval growth rates for the Cádiz population suggest some growth limitation at 24°C (Šargač et al. 2022). For Helgoland, the optimal temperature is in the range 15°C–18°C (Šargač et al. 2022; Torres and Giménez 2020). For the upper thermal limit, we did not find differences among the three tested populations; all had very low survival rates at 27°C. In the studied populations from North America, no survival to megalopa was reported at temperatures higher than 22.5°C (25°C was the next higher tested temperature). This is substantially lower than the highest temperature that allowed survival in Vigo, Bergen, and Trondheim, and while we do not have the upper limit for Cádiz, the high survival at 24°C suggests that the higher limit should be above 25°C. Comparing all *C. maenas* populations with available data, we found a decrease in the temperature that yields highest survival rates with latitude (Figure 6a), which could have been attributed to an adaptive response (or long‐term acclimation to habitat conditions) to the respective thermal regime. However, this trend was not retained in the best models where the predictors were average temperature or season length (Figure 6d,g). In addition, the statistical models fitted to larvae from the Norwegian populations predict a shorter duration of development at 12°C and 15°C than the model fitted to the Vigo population, hinting at an adaptation to the local thermal regime and shorter phenological windows in higher latitudes (Figure 6g–i).

The integration of physiological and population responses, given by total production (survival × carbon content), resulted in a drop above 21°C (in all populations). Therefore, our results suggest that increased temperature above 21°C in the field possibly results in a reduction of carbon fluxes from the pelagic to the benthic habitat, as mediated by larval transport of *C. maenas* (with other factors such as current patterns kept constant). Since meroplanktonic larvae of benthic predators serve as a food source for pelagic predators, they contribute to bentho‐pelagic coupling. While *C. maenas* larvae can temporarily comprise up to 70% of individual mesozooplankters in the German Bight, comparisons of dry mass and carbon contents with field data are difficult because most plankton studies report biomass/carbon content by size fractions rather than species and life stage (Harms et al. 1994). But the importance of crab larvae has been demonstrated. In the Bristol Channel, Celtic Sea, for example, meroplankton exceeded holoplankton and made up 58% of total omnivorous zooplankton biomass, with decapod larvae being the dominating group (Williams and Collins 1986). In Ria de Arosa, Northern Spain, > 90% of zooplankton biomass are zoeas of the anomuran crab *Pisidia longicornis* (Tenore et al. 1982). In Auke Bay, Alaska, meroplankton made up 5%–40% of total zooplankton biomass during the spring bloom (Coyle and Paul 1990).

### 4.3 Conclusions

In synthesis, both our experiment and a comparison to literature data add to a growing body of literature investigating *C. maenas* as a model system for the study of climate change impacts on life history, physiology, and distribution of marine invertebrates with complex life cycles and show that the duration of development at low temperatures (e.g., 12°C) is shorter in the northern populations. This might have an adaptive value for Norwegian populations, contingent on the actual pattern of temperatures experienced during the larval phase. The population from Cádiz, southern Spain, showed slightly increased survival at higher temperatures, as shown in the literature, but we cannot provide experimental evidence of an increased upper limit of thermal tolerance with the population from Vigo, Northern Spain, which seems more similar to western and northern European populations. Further range expansions into the European Arctic are likely to be driven solely by increasing temperatures unless populations from the Arctic show increased tolerance to low temperatures. Range contractions associated with limited larval tolerance to high temperature appear likely in the face of increasing summer temperatures and marine heatwaves unless other populations located at the southern limit show increased thermal tolerance.

## Author Contributions

**Jan Phillipp Geißel:** conceptualization (supporting), data curation (lead), formal analysis (lead), investigation (lead), methodology (lead), visualization (lead), writing – original draft (lead), writing – review and editing (equal). **Noé Espinosa‐Novo:** conceptualization (supporting), data curation (lead), formal analysis (lead), investigation (lead), methodology (lead), visualization (lead), writing – original draft (lead), writing – review and editing (equal). **Luis Giménez:** conceptualization (lead), formal analysis (supporting), funding acquisition (supporting), investigation (supporting), methodology (supporting), supervision (equal), visualization (supporting), writing – review and editing (equal). **Nicole Aberle:** investigation (supporting), project administration (supporting), resources (supporting), writing – review and editing (equal). **Gro I. van der Meeren:** investigation (supporting), resources (supporting), writing – review and editing (equal). **Steffen Harzsch:** conceptualization (lead), funding acquisition (lead), project administration (lead), resources (supporting), supervision (lead), writing – review and editing (equal). **Maarten Boersma:** funding acquisition (supporting), resources (supporting), supervision (lead), writing – review and editing (equal). **Gabriela Torres:** conceptualization (lead), formal analysis (supporting), funding acquisition (lead), investigation (supporting), methodology (supporting), project administration (lead), resources (lead), supervision (lead), visualization (supporting), writing – review and editing (equal).

## Disclosure

*Statement on inclusion*: Our study involved authors, scientists, and citizen‐scientists from a number of different countries and institutions, including scientists based in Norway where a big part of this study was carried out. We did not include scientists from Vigo, Spain (one of the sites where animals were obtained for this study), because these animals were obtained as part of a research visit from N.E.‐N. and G.T. to the ECIMAT (Estación de Ciencias Mariñas de Toralla, Universidad de Vigo) funded by Transnational Access ASSEMBLE+ (see Funding). All authors were involved early on with the research, study design, and collection of animals to ensure that local knowledge and perspectives were considered. Furthermore, literature published by scientists from these regions and all the distribution ranges of the model species were cited.

*Human or animal rights*: The research presented in this paper complies with national (Germany) and international laws (guidelines from the directives 2010/63/EU of the European parliament and of the Council of 22nd September 2010) on the protection of animals used for scientific purposes. In addition, this research complies with the European regulation (N°511/2014) regarding access to genetic resources and the fair and equitable sharing of benefits arising from their utilisation (Nagoya protocol). Authorisation to access Spanish genetic resources ( *Carcinus maenas* ): Ref. ESNC68, CSV: GEN‐8e2b‐06b0‐12a7‐b306‐7d90‐4683‐1db3‐ed89 (https://sede.administracion.gob.es/pagSedeFront/servicios/consultaCSV.htm), granted by the Dirección General de Patrimonio Natural de la Xunta de Galicia.

## Conflicts of Interest

The authors declare no conflicts of interest.

## Supporting information


Data S1.

## Data Availability

All data for this paper will be available from PANGAEA Data Publisher https://www.pangaea.de/.
