# Supplementary material for: Larval Physiological Responses to Temperature Across the European Distribution Range of a Global Invader at Home: The Shore Crab Carcinus maenas
Source: Ecol Evol. 2025 Jun 13;15(6):e71587. doi: 10.1002/ece3.71587 (PMC12166129; doi:10.1002/ece3.71587)
Supplement: Supplementary file 1 — Data S1. [file ECE3-15-e71587-s001.docx]

SUPPLEMENTARY MATERIAL

**MATERIALS & METHODS**

**Table S1.** Correlation coefficients and p-values for the analysis of correlations between carapace size of mother (CW) and response variables of *Carcinus maenas* considering larvae from Vigo, Bergen and Trondheim reared at different temperatures. P-values approximated to two digits, correlation coefficients approximated to four digits.

| Response | Temperature (°C) | Correlation coefficient | R^2^ | p-value |
| --- | --- | --- | --- | --- |
| Survival to Zii *vs* CW | 12 | 0.0903 | 0.082 | 0.74 |
|  | 15 | 0.1324 | 0.0175 | 0.63 |
|  | 18 | 0.3475 | 0.1208 | 0.19 |
|  | 21 | 0.1707 | 0.0291 | 0.53 |
|  | 24 | 0.3409 | 0.1162 | 0.20 |
| Duration of development to Zii *vs* CW | 12 | -0.1790 | 0.0320 | 0.51 |
|  | 15 | -0.2310 | 0.0534 | 0.41 |
|  | 18 | -0.3865 | 0.1494 | 0.14 |
|  | 21 | -0.2768 | 0.0411 | 0.45 |
|  | 24 | -0.2066 | 0.0427 | 0.34 |
| Carbon content at hatch *vs* CW | 12, 15 | -0.3610 | 0.1303 | 0.19 |
| Dry mass at hatch *vs* CW | 12, 15 | -0.1657 | 0.0275 | 0.54 |
| Nitrogen content at hatch *vs* CW | 12, 15 | -0.1964 | 0.0385 | 0.15 |
| Survival to M *vs* CW | 12 | 0.2006 | 0.0402 | 0.51 |
|  | 15 | -0.0374 | 0.0014 | 0.90 |
|  | 18 | 0.2786 | 0.0776 | 0.36 |
|  | 21 | 0.1651 | 0.0273 | 0.59 |
|  | 24 | 0.2214 | 0.0490 | 0.47 |
| Duration of development to M *vs* CW | 12 | 0.2262 | 0.0512 | 0.53 |
|  | 15 | 0.3815 | 0.1455 | 0.22 |
|  | 18 | -0.2508 | 0.0629 | 0.41 |
|  | 21 | 0.1278 | 0.0163 | 0.69 |
|  | 24 | 0.1426 | 0.0203 | 0.68 |
| Carbon content of M *vs* CW | 12 | 0.1899 | 0.0361 | 0.60 |
|  | 15 | -01661 | 0.0276 | 0.61 |
|  | 18 | -0.5218 | 0.2723 | 0.07 |
|  | 21 | 0.2195 | 0.0482 | 0.49 |
|  | 24 | -0.4480 | 0.2007 | 0.17 |
| Dry mass of M *vs* CW | 12 | 0.0795 | 0.0063 | 0.83 |
|  | 15 | -0.3186 | 0.1015 | 0.31 |
|  | 18 | -0.6126 | 0.3753 | 0.03 |
|  | 21 | 0.0688 | 0.0047 | 0.83 |
|  | 24 | -0.5948 | 0.3537 | 0.05 |
| Nitrogen content of M *vs* CW | 12 | 0.1136 | 0.0129 | 0.75 |
|  | 15 | -0.1216 | 0.0148 | 0.71 |
|  | 18 | -0.6026 | 0.3631 | 0.03 |
|  | 21 | 0.1327 | 0.0176 | 0.68 |
|  | 24 | -0.5125 | 0.2626 | 0.11 |
| Instantaneous growth rates to M *vs* incubation time (carbon-based) | 12 | 0.4633 | 0.2146 | 0.18 |
|  | 15 | -0.1824 | 0.0333 | 0.57 |
|  | 18 | -0.1328 | 0.0176 | 0.67 |
|  | 21 | 0.4219 | 0.1780 | 0.17 |
|  | 24 | -0.1900 | 0.0361 | 0.58 |
| Instantaneous growth rates to M *vs* incubation time (dry mass-based) | 12 | 0.3050 | 0.0930 | 0.39 |
|  | 15 | -0.2313 | 0.0535 | 0.47 |
|  | 18 | -0.1422 | 0.2020 | 0.64 |
|  | 21 | 0.3022 | 0.0913 | 0.34 |
|  | 24 | -0.3322 | 0.1104 | 0.32 |
| Instantaneous growth rates to M *vs* incubation time (nitrogen- based) | 12 | 0.3798 | 0.1443 | 0.28 |
|  | 15 | -01626 | 0.0264 | 0.61 |
|  | 18 | -0.1559 | 0.0243 | 0.61 |
|  | 21 | 0.3983 | 0.1586 | 0.20 |
|  | 24 | -0.2342 | 0.0548 | 0.49 |

**Table S2.** Correlation coefficients and p-values for the analysis of correlations between incubation time and response variables of *Carcinus maenas* considering larvae from Vigo, Bergen and Trondheim reared at different temperatures. P-values approximated to two digits, correlation coefficients approximated to four digits.

| Response | Temperature (°C) | Correlation coefficient | R^2^ | p-value |
| --- | --- | --- | --- | --- |
| Dry mass at hatch *vs* incubation time | 12, 15 | 0.0008 | 0.0000 | 1.00 |
| Survival to M *vs* incubation time | 12 | -0.0849 | 0.0402 | 0.51 |
|  | 15 | 0.3742 | 0.0014 | 0.90 |
|  | 18 | 0.4334 | 0.1878 | 0.11 |
|  | 21 | 0.5157 | 0.2659 | 0.05 |
|  | 24 | 0.3451 | 0.1191 | 0.21 |
| Duration of development to M *vs* incubation time | 12 | -0.0270 | 0.0007 | 0.93 |
|  | 15 | -0.0968 | 0.0094 | 0.74 |
|  | 18 | -0.5111 | 0.2612 | 0.05 |
|  | 21 | -0.1666 | 0.0278 | 0.57 |
|  | 24 | -0.1466 | 0.0215 | 0.63 |
| Carbon content of M *vs* incubation time | 12 | -0.4057 | 0.1646 | 2.12 E-05 |
|  | 15 | 0.1468 | 0.0215 | 0.01 |
|  | 18 | 0.0807 | 0.0065 | 0.18 |
|  | 21 | 0.0733 | 0.0054 | 0.27 |
|  | 24 | 0.0784 | 0.0062 | 0.46 |
| Dry mass of M *vs* incubation time | 12 | -0.2856 | 0.0816 | 0.003 |
|  | 15 | -0.0321 | 0.0010 | 0.57 |
|  | 18 | -0.1442 | 0.0208 | 0.02 |
|  | 21 | -0.1599 | 0.0256 | 0.01 |
|  | 24 | -0.0785 | 0.0062 | 0.46 |
| Nitrogen content of M *vs* incubation time | 12 | -0.2787 | 0.0777 | 0.004 |
|  | 15 | -0.0707 | 0.0050 | 0.21 |
|  | 18 | -0.0276 | 0.0008 | 0.65 |
|  | 21 | -0.0452 | 0.0020 | 0.49 |
|  | 24 | -0.0731 | 0.0053 | 0.49 |
| Instantaneous growth rates to M *vs* incubation time (carbon-based) | 12 | -0.1793 | 0.0322 | 0.07 |
|  | 15 | 0.0754 | 0.0057 | 0.19 |
|  | 18 | 0.1324 | 0.0175 | 0.03 |
|  | 21 | 0.0448 | 0.0020 | 0.50 |
|  | 24 | 0.0550 | 0.0030 | 0.60 |
| Instantaneous growth rates to M *vs* incubation time (dry mass-based) | 12 | -0.1316 | 0.0173 | 0.19 |
|  | 15 | -0.0513 | 0.0026 | 0.37 |
|  | 18 | -0.0079 | 0.0001 | 0.90 |
|  | 21 | -0.1212 | 0.0147 | 0.07 |
|  | 24 | -0.0856 | 0.0073 | 0.42 |
| Instantaneous growth rates to M *vs* incubation time (nitrogen- based) | 12 | -0.0963 | 0.0093 | 0.33 |
|  | 15 | 0.0219 | 0.0005 | 0.70 |
|  | 18 | 0.0533 | 0.0028 | 0.38 |
|  | 21 | -0.0445 | 0.0020 | 0.50 |
|  | 24 | -0.0632 | 0.0040 | 0.55 |


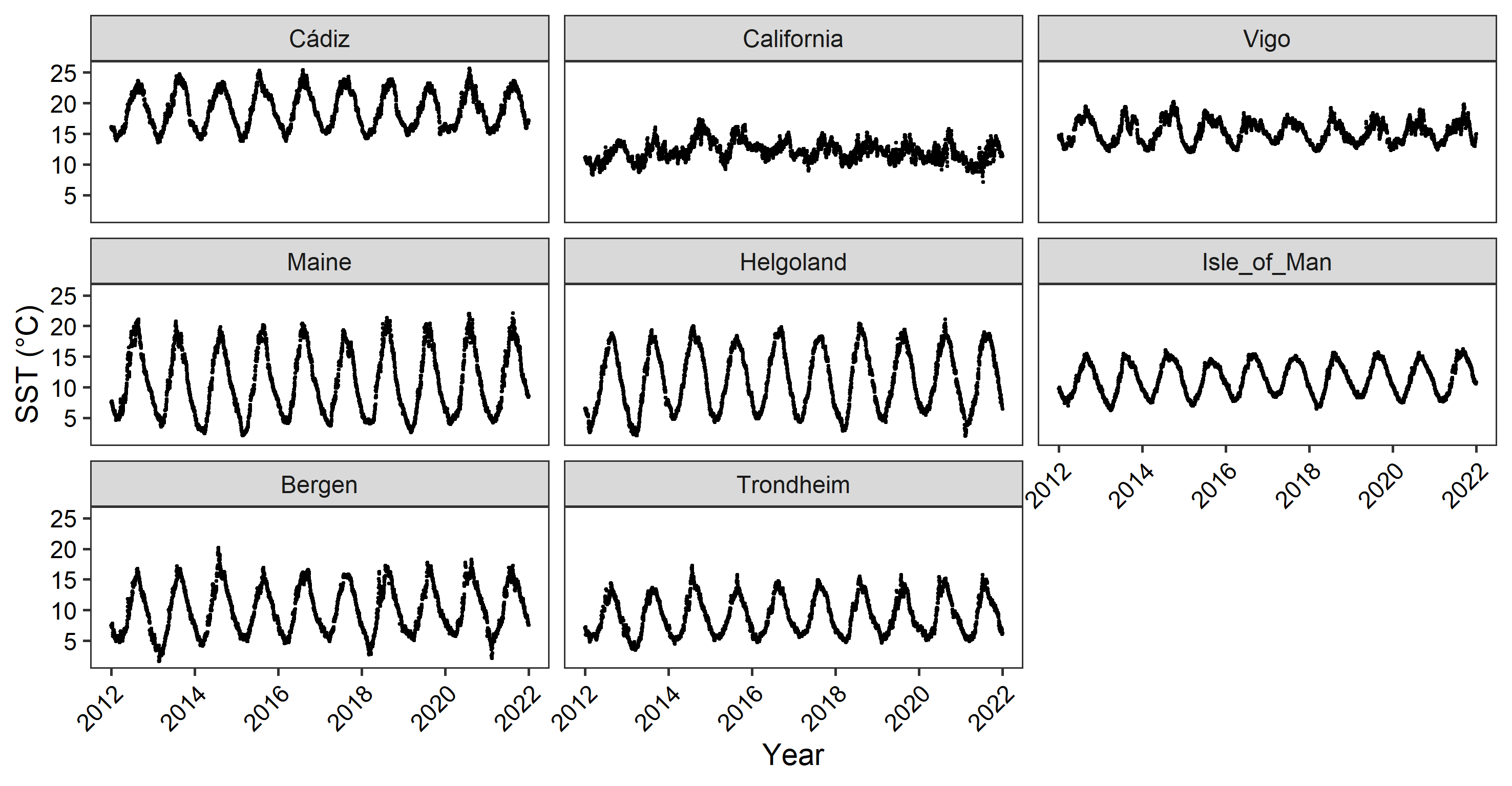


**Figure S1.** Daily sea surface temperatures (SST, °C) for the sites investigated in this study for the years 2012 to 2022 using E.U. Copernicus Marine Service Information; <https://doi.org/10.48670/moi-00165>.


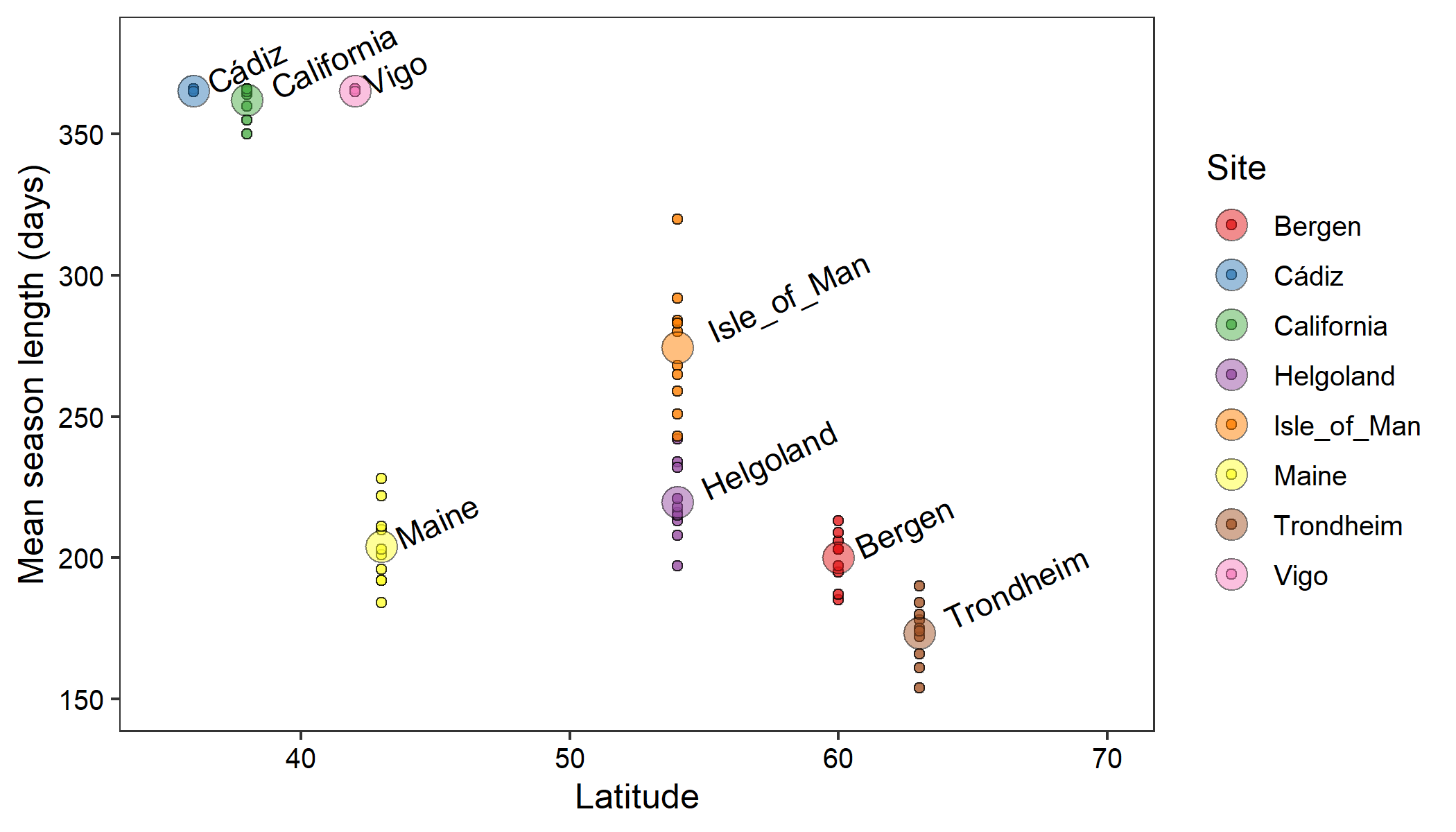


**Figure S2.** Main season length (number of consecutive days with temperatures between 9 and 27 °C) for the sites investigated in this study for the years 2012 to 2022. Based on daily sea surface temperatures (SST, °C) using E.U. Copernicus Marine Service Information; <https://doi.org/10.48670/moi-00165>. Large circles indicate means per site over the entire period, small circles indicate the means per year for the respective sites. Colours indicate sites.

**RESULTS**

**Table S3.** Model selection for survival to megalopa of *Carcinus maenas* considering larvae from Vigo, Bergen, and Trondheim, reared at different temperatures. Data were analysed in the logistic and logarithmic scales. Model selection was performed using corrected Akaike information criteria (AICc). Symbols: ♀ female of origin, T temperature, P population of origin. Highlighted in bold: the best overall model, for both the random and fixed term.

| **Model selection:** | | **Vigo - Bergen - Trondheim** | |
| --- | --- | --- | --- |
|  |  | **Raw** | **Log** |
| **Random (REML)** | |  |  |
| ♀:T:P |  | -476 | 938 |
| ♀:T |  | **-504** | **913** |
| ♀:P |  | -335 | 1050 |
| ♀ |  | -340 | 1046 |
| **Fixed (ML)** | |  |  |
| P:T | | -588 | 874 |
| P+T | | -607 | 888 |
| T | | **-610** | **870** |
| P | | -538 | 978 |
| Null | | -542 | 974 |

**Table S4.** Model selection for duration of development to megalopa of *Carcinus maenas* considering larvae from Vigo, Bergen and Trondheim, reared at different temperatures. Data were analysed using two modelling approaches: a) in model 1, temperature and population were included as fixed factors and in b) model 2, temperature was included as a continuous variable. Model selection was performed using corrected Akaike information criteria (AICc). Symbols: ♀ female of origin, T temperature, P population of origin. Highlighted in bold: the best overall model, for both the random and fixed term.

1. **Model 1**

| **Model selection:** | | **Vigo - Bergen - Trondheim** | |
| --- | --- | --- | --- |
|  |  | **Model 1** | |
| **Random (REML)** | |  |  |
| ♀:T:P |  | 4756 | |
| ♀:T |  | **4740** | |
| ♀:P |  | 4744 | |
| ♀ |  | 4742 | |
| **Fixed (ML)** | |  |  |
| P:T | | **4750** | |
| P+T | | 4771 | |
| T | | 4771 | |
| P | | 5052 | |
| Null | | 5053 | |

1. **Model 2**

| **Model selection:** | | | **Vigo - Bergen - Trondheim** | |
| --- | --- | --- | --- | --- |
|  |  |  | **Model 2** | |
| **Random (REML)** | | |  |  |
| ♀:T:P |  | | **-1526** | |
| ♀:T |  | | -1255 | |
| **Fixed (ML)** |  | | | |
| P:T |  | **-1546** | | |
| P+T |  | 444 | | |


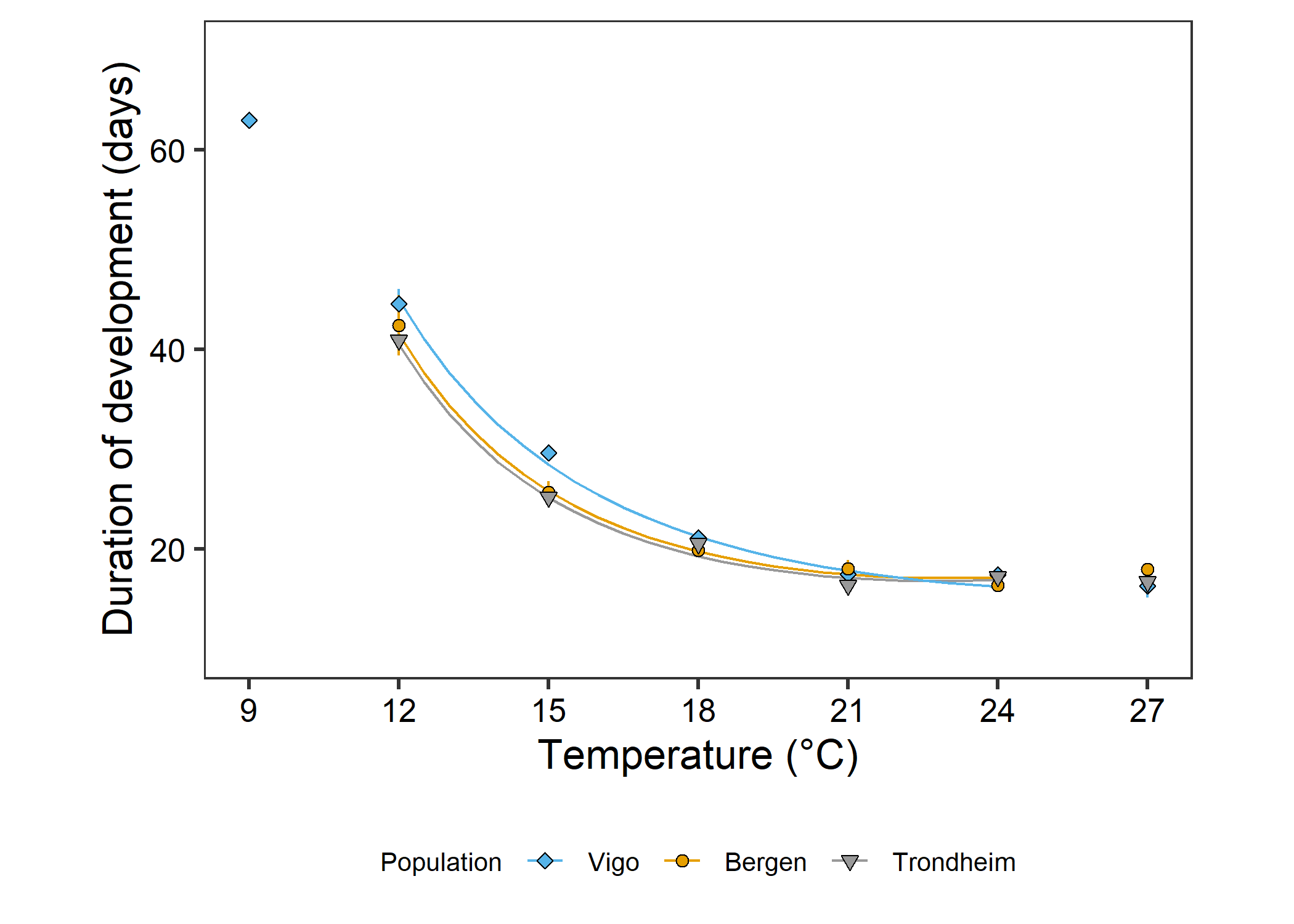


**Figure S3.** Average duration of development from hatching to megalopa of *Carcinus maenas* larvae reared under different temperatures, from three populations: Vigo, Bergen, and Trondheim. Data presented as mean values ± SE of larvae produced by different females of origin. Lines show predicted developmental durations based on maximum likelihood (DD = a * T^b^) with a being constant and b being a log(T) parameter depending on population. Note that at 9 and 27 °C the sample size was limited to include these temperatures in the model. Symbols as in Fig. 2.

**Table S5.** Model selection for dry mass (DM), carbon (C) and nitrogen (N) content per individual and instantaneous growth rates to megalopa of *Carcinus maenas* in terms of dry mass (IgDW), carbon (IgC) and nitrogen (IgN); considering larvae from Vigo, Bergen, and Trondheim, reared at different temperatures. Model selection was performed using corrected Akaike information criteria (AICc). Symbols: ♀ female of origin, T temperature, P population of origin. Highlighted in bold: the best overall model, for both the random and fixed term. For growth based on carbon, the full factorial model did not reach convergence.

| Random (REML) | | AICc | | | | | | | | | | |
| --- | --- | --- | --- | --- | --- | --- | --- | --- | --- | --- | --- | --- |
| Term | | DW | C | | N | | IgDW | IgC | | IgN | | C/N |
|  | ♀:T:P | 8225 | 6269 | | 3058 | | -5410 | **-** | | -5436 | | 1254 |
|  | ♀:T | **8209** | **6253** | | **3044** | | **-5428** | **-5362** | | **-5455** | | **1233** |
|  | ♀:P | 8227 | 6274 | | 3054 | | -5419 | -5348 | | -5442 | | 1249 |
|  | ♀ | 8223 | 6270 | | 3050 | | -5423 | -5352 | | -5446 | | 1245 |
| Fixed (ML) | |  | |  | |  | | |  | |  | |
|  | Full factorial | 8266 | 6285 | | 3023 | | -5574 | -5505 | | -5601 | | **1187** |
|  | P+T | **8254** | **6278** | | **3017** | | -5571 | -5503 | | -5599 | | 1188 |
|  | T | 8268 | 6289 | | 3031 | | **-5572** | **-5504** | | **-5601** | | 1198 |
|  | P | 8278 | 6295 | | 3035 | | -5381 | -5316 | | -5409 | | 1211 |
|  | Null model | 8288 | 6303 | | 3045 | | -5382 | -5318 | | -5412 | | 1218 |


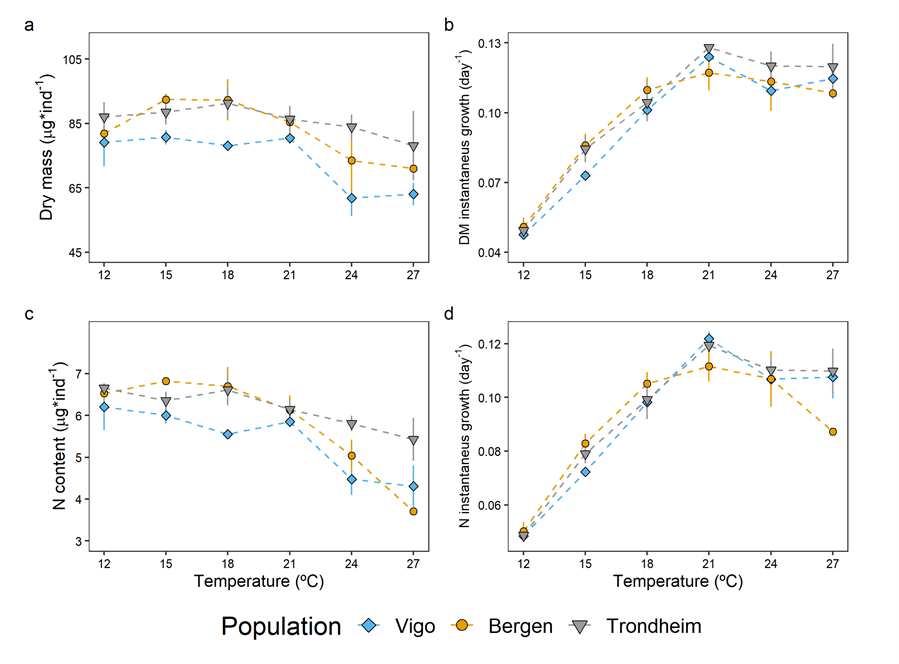


**Figure S4**. (a) Average dry mass. (b) Average growth rates in terms of dry mass. (c) Average nitrogen content. (d) Average growth rates in terms of nitrogen content. Data represented in the graphs correspond to larvae of *Carcinus maenas* reared from hatching to megalopa under different temperatures, from three populations: Vigo, Bergen, and Trondheim. Data presented as mean values ± SE of larvae produced by different females of origin. Symbols as in Fig. 2.


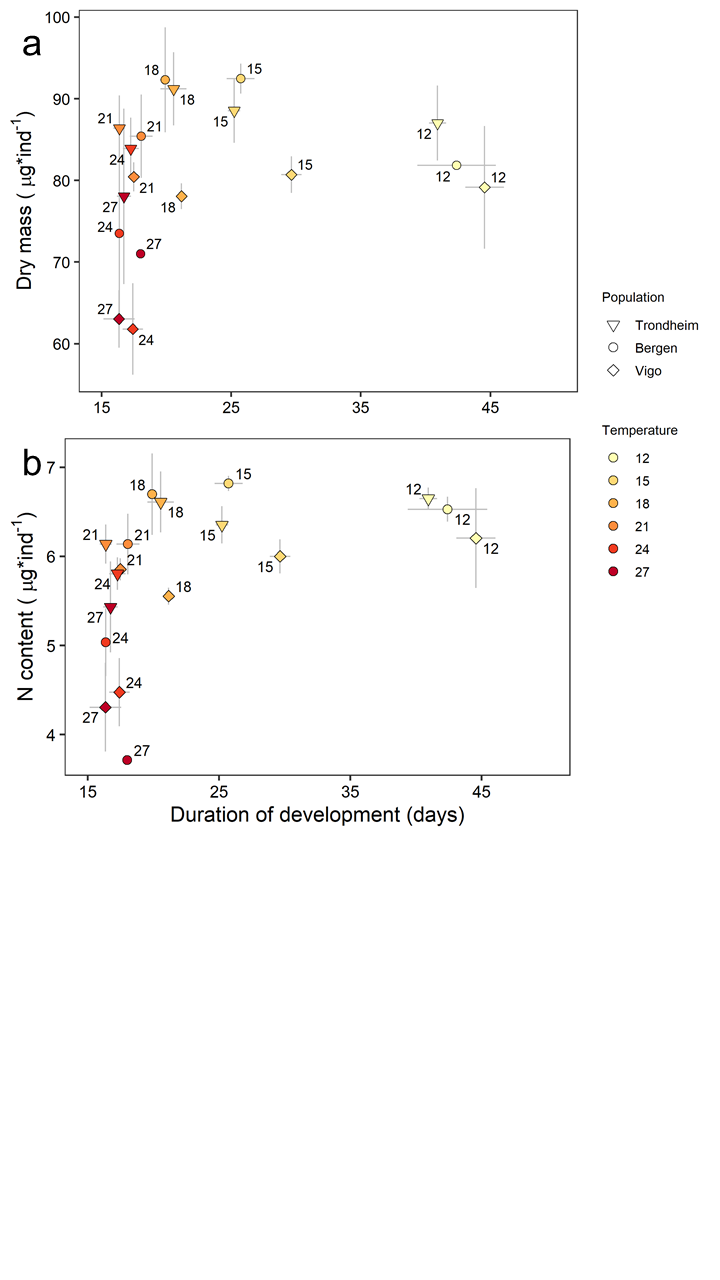


**Figure S5**. Integrated responses of dry mass (a) and nitrogen content (b) and duration of development of larvae of *Carcinus maenas* reared under different temperatures, form hatching to megalopa, from three populations: Vigo, Bergen, and Trondheim. Data presented as mean values ± SE of larvae produced by different females of origin. Symbols: Vigo is represented with diamonds, Bergen with circles, and Trondheim with triangles. Colours and labels indicate temperature.


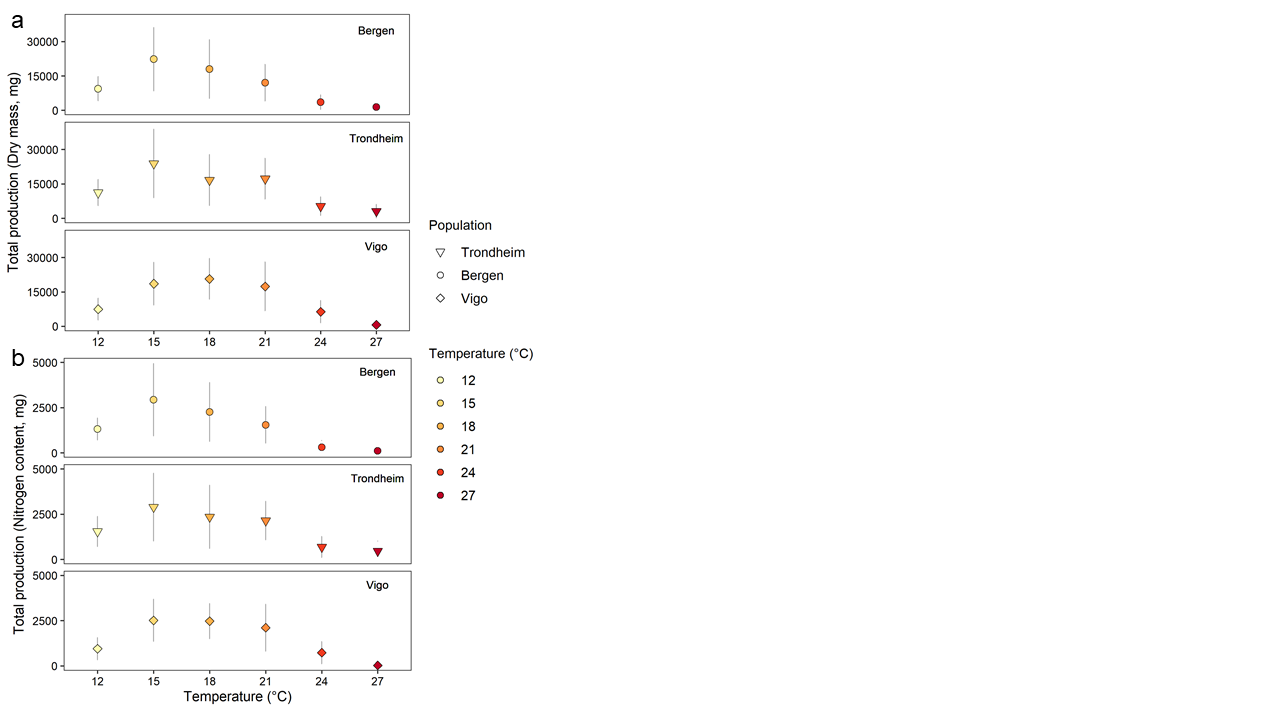


**Figure S6.** Effects of temperature on total production, measured as the number of survivors * dry mass (a) and nitrogen content (b) of larvae of *Carcinus maenas* reared under different temperatures, from hatching to megalopa, from larvae originating from females from three populations: Vigo, Bergen, and Trondheim. Data presented as mean values ± SE among larvae from different females within each population (n= 6 for Vigo; n= 4 for Bergen and n= 5 for Trondheim). Symbols: Vigo is represented with diamonds, Bergen with circles, and Trondheim with triangles. Symbols as in Fig. S3.
